# Elevation of Glucose 6-Phosphate Dehydrogenase Activity Induced by Amplified Insulin Response in Low Glutathione Levels in Rat Liver

**DOI:** 10.1155/2016/6382467

**Published:** 2016-08-15

**Authors:** Misako Taniguchi, Nobuko Mori, Chizuru Iramina, Akira Yasutake

**Affiliations:** ^1^Department of Nutrition Sciences, Nakamura Gakuen University, Fukuoka 814-0198, Japan; ^2^Department of Biomedical Laboratory Sciences, Faculty of Life Sciences, Kumamoto University, 4-24-1 Kuhonji, Kumamoto 862-0976, Japan; ^3^Kumamoto University Graduate School of Science and Technology, 2-39-1 Kurokami, Kumamoto 860-8555, Japan

## Abstract

Weanling male Wistar rats were fed on a 10% soybean protein isolate (SPI) diet for 3 weeks with or without supplementing 0.3% sulfur-containing amino acids (SAA; methionine or cystine) to examine relationship between glutathione (GSH) levels and activities of NADPH-producing enzymes, glucose 6-phosphate dehydrogenase (G6PD) and malic enzyme (ME), in the liver. Of rats on the 10% SPI diet, GSH levels were lower and the enzyme activities were higher than of those fed on an SAA-supplemented diet. Despite the lower GSH level, *γ*-glutamylcysteine synthetase (*γ*-GCS) activity was higher in the 10% SPI group than other groups. Examination of mRNAs of G6PD and ME suggested that the GSH-suppressing effect on enzyme induction occurred prior to and/or at transcriptional levels. Gel electrophoresis of G6PD indicated that low GSH status caused a decrease in reduced form and an increase in oxidized form of the enzyme, suggesting an accelerated turnover rate of the enzyme. In primary cultured hepatocytes, insulin response to induce G6PD activity was augmented in low GSH levels manipulated in the presence of buthionine sulfoximine. These findings indicated that elevation of the G6PD activity in low GSH levels was caused by amplified insulin response for expression of the enzyme and accelerated turnover rate of the enzyme molecule.

## 1. Introduction

As a protection mechanism against oxidative damage, tissues are equipped with anti-oxidative-reducing thiol compounds such as glutathione (GSH) and metallothionein (MT), and their concentrations are elevated under oxidative stress. In fact, after treating rats with N-nitrosodimethylamine, which forms reactive oxygen species, GSH and MT levels elevated significantly [1, 2]. Concentrations of these substances in tissues are readily affected by dietary conditions, which then affect the protective ability against oxidative damage. GSH levels in the liver were regulated by the amount of sulfur-containing amino acids (SAA, methionine and cystine) in a diet [1], while MT levels were induced by heavy metals [3]. In addition to GSH and MT, NADPH plays an important role as a defense mechanism by providing a reducing-radical against oxidative stress. NADPH is produced primarily by glucose 6-phosphate dehydrogenase (G6PD) and malic enzyme. G6PD is a key enzyme of the pentose phosphate pathway, and its activity is altered by complex interactions of dietary components and hormones. A high carbohydrate or low fat diet increased hepatic stimulated induction of G6PD activity in rat liver [4, 5]. Insulin [6–9] and thyroid hormone [10] elevated induction of G6PD, while glucagon suppressed it [11]. G6PD activity varied by dietary protein type [12], but the mechanism that modulates G6PD activity by the protein has not been elucidated. In Hela cell clones, G6PD expression was enhanced under GSH restricted states [13]. GSH is known as a cysteine reservoir in the liver [14], and intracellular GSH levels readily declined when rats received an SAA-deficient diet [1, 2]. In intact tissues, G6PD molecules exist in three major dimeric forms: fully reduced, partly oxidized, and fully oxidized forms, and different turnover rates were indicated for the three forms [15–17]. In this study, we attempted to elucidate the mechanism that regulates G6PD activity linked with hepatic GSH levels, using a diet with 10% soybean protein isolate (SPI) that has a low SAA content. To amplify SAA content, a 10% SPI diet was supplemented with 0.3% cystine or methionine. Effects of a low SAA diet on ME activity were also examined in comparison to G6PD. In addition, the direct effect of insulin response on G6PD induction at different GSH concentrations was examined using isolated primary cultured hepatocytes.

## 2. Materials and Methods

### 2.1. Chemicals

Glutathione (both reduced and oxidized forms) and glucose 6-phosphate were purchased from Boehringer Mannheim GmbH (Mannheim, Germany). NADP and NADPH were purchased from Oriental Yeast Co., Ltd. (Tokyo, Japan). *γ*-Glutamylglutamic acid (used as an internal standard in HPLC analysis), *γ*-glutamylcysteine, and insulin were purchased from Sigma Chemical Co. (St Louis, MO, USA). The materials used for isolation and cell culture were as reported previously by Yoshimoto et al. [8]. All other chemicals were of reagent grade.

### 2.2. Animals and Diet

As protein sources, SPI, provided from Fuji Oil Co., Ltd. (Osaka, Japan), was used. SPI consisted of 91.1% crude protein, 4% salts, and 3.5% carbohydrates. Amino acid composition of SPI was analyzed in the research center of Fuji Oil Co., and 1.4 g of methionine and 1.3 g of cystine were contained per 16 g of nitrogen as described in our previous paper [18]. Diet was prepared according to the recommendation of AIN 93 [19] as shown in Table 1. To examine the effects of SAA supplementation, 0.3% cystine or methionine was added in 10% SPI. As a whole, 3 dietary groups were used in the present study: 10% SPI, 10% SPI + 0.3% cystine, and 10% SPI + 0.3% methionine. Weanling male Wistar rats (Charles River, Japan; body weight of 55 ± 3 g, 6 in each group) were housed in individual cages and maintained in a temperature-controlled room at 20–22°C with a controlled 12 h cycle of light and darkness. Diets and drinking water were given ad libitum during the experiment. After the feeding period for 3 weeks, rats were sacrificed from 09:00 to 10:30 under deep anesthetization with diethylether. Four pieces of liver samples were obtained from each rat for the assays of GSH and MT, for the determination of enzyme activities and mRNA levels. All procedures were conducted in accordance with the* Guide for the Care and Use of Laboratory Animals* of Nakamura Gakuen University.

*Note.* The above animal experiment was carried out in 1998 when diethylether anesthesia could be used without any regulation.

### 2.3. Assays of GSH and MT

For glutathione assay, a portion (ca. 0.5 g) of the liver was immediately homogenized in ice-cold 5% perchloric acid (PCA) containing 1 mM EDTA. After centrifugation at 2,500 rpm for 10 min, reduced (GSH) and oxidized glutathione (GSSG) in the supernatant fraction were determined by HPLC after derivatizing with dinitrobenzene fluoride [20]. For MT analysis, portions of liver (0.5 g) were kept at −80°C until use. The liver sample was homogenized (20%, w/v) in ice-cold 1.15% KCl using a Polytron homogenizer (Kinematica GmbH, Littau, Switzerland) under N_2_ atmosphere. An aliquot of the homogenate was subjected to MT assay according to the procedure of Naganuma et al. [21] with a slight modification using nonradioactive HgCl_2_ [22]. Briefly, the homogenate was treated successively with diethyl maleate and 10 mM CdCl_2_ and then heated at 95°C for 5 min to precipitate high-molecular weight proteins. Following cooling and centrifugation, the supernatant was successively treated with 5 mM HgCl_2_, 1 mM ovalbumin, and 12.5% trichloroacetic acid. After centrifugation, the supernatant was filtered through a membrane of 0.22 *μ*m pore diameter (Ultrafree C3, Merck Millipore, Darmstadt, Germany) to afford Hg-MT samples. MT levels were expressed as the amount of mercury bound to thionein molecules after Hg analysis of the Hg-MT samples. Hg levels in the final preparations were determined by the oxygen combustion-gold amalgamation method [23] using an atomic absorption mercury detector MD-A (Nippon Instruments Co., Ltd., Osaka, Japan).

### 2.4. Assay of Enzyme Activity

A portion of the liver (1-2 g) was homogenized in 5 vol. of an ice-cold homogenizing buffer, 0.25 M sucrose solution containing 5 mM Tris-HCl buffer (pH 7.4), and 0.1 mM EDTA. After centrifugation, the upper layer was used for determination of enzyme activities. G6PD and ME activities were determined according to the method of Lee [24] and Ochoa [25], respectively. One unit of G6PD or ME activity was defined as the amount of enzyme that catalyzed oxidation of 1 *μ*mol of glucose 6-phosphate or malate per min at 25°C.

*γ*-Glutamylcysteine synthetase (*γ*-GCS) activity was determined by the method of Nardi et al. [26] with a slight modification. The supernatant described above was added to the reaction mixture consisting of 6 mM ATP, 50 mM KCl, 6 mM dithiothreitol, 20 mM MgCl_2_, 3 mM L-cystine, and 15 mM glutamic acid in 0.1 M Tris-HCl buffer (pH 8.2) and then incubated at 37°C for 10 min. The reaction was terminated by adding 110 *μ*L of 5% PCA and centrifuged to afford a supernatant fraction. *γ*-Glutamylcysteine formed in the mixture was derivatized with dinitrofluorobenzene and analyzed as described above for GSH determination using HPLC [20]. One unit of *γ*-GCS activity was defined as the amount of enzyme that catalyzes to form 1 *μ*mol of *γ*-glutamylcysteine per min at 37°C.

Protein was determined by the method of Lowry et al. [27], and the enzyme activities were expressed as mU/mg protein.

### 2.5. mRNA

Total RNA was isolated from fresh liver sample (ca. 0.5 g) obtained above by a modified acid guanidinium-phenol extraction method of Chomczynski and Sacchi [28], using a commercially available RNA isolation reagent ISOGEN (Nippon Gene, Tokyo, Japan) according to the protocol recommended by the manufacturer. Yield and purity were determined by absorbance at 260 nm and absorbance ratio* A*_260_/*A*_280_ (1.7–2.0). RNA samples (10 *μ*g each in 0.2 mL of H_2_O/20x SSC/37% formaldehyde, 2 : 1 : 1) were incubated at 65°C for 15 min and then loaded in the slot-blot apparatus (Bio-Rad Japan, Tokyo) onto a Clear Blot Membrane-N (ATTO Co., Tokyo, Japan). G6PD and ME mRNAs on the membrane were analyzed by a solution hybridization procedure described by Hamilton et al. [29] using *α*-^32^P-dCTP and DNA probes for each enzyme. The probes were prepared by RT-PCR method using rat liver RNA and the following primer pairs:

 G6PD
   sense primer: 5′-GTCCTCTATGTGGAGAATGA-3′
   antisense primer: 5′-TCTTGGTCATCATCTTGGTA-3′
 ME
   sense primer: 5′-TCCAGGTCCTTAGAGTAATT;
   antisense primer: 5′-CATGCGTTAAGAACTGAAGA-3′

 The mRNA levels were estimated by comparison with *β*-actin mRNA level.

### 2.6. Gel Electrophoresis

Distribution of fully reduced, partly oxidized, and fully oxidized forms of G6PD in rat liver was determined using electrophoresis according to the method by Martins et al. [17]. Cytosol samples from the respective dietary groups containing equivalent G6PD activity (5 mU) were applied on a polyacrylamide gel and electrophoresed within 3 hr of preparation using a 1.5 mm thick slab gel containing 10% (w/v) acrylamide and 0.26% (w/v) bisacrylamide polymerized in the presence of 0.017% (w/v) ammonium persulphate and 0.033% (v/v) tetramethylene diamine. The gel was stained for G6PD activity in 50 mM Tris-HCl (pH 8.0) containing 0.58 mM glucose 6-phosphate, 0.13 mM NADP^+^, 10 mM MgCl_2_, 33 *μ*M phenazine methosulphate, and 0.1 mM nitroblue tetrazolium. After incubation at 4°C for 16 hr, the reaction was terminated by immersing the gel in 7% acetic acid. The gel was dried, and intensities of the enzyme fractions were scanned using computing densitometer (ACD-18; Gelman Science, Inc., Pensacola, FL, USA).

### 2.7. Cell Culture

Liver parenchymal cells were isolated from male Wistar rat (6 weeks of age) fed with laboratory chow diet (CE-2) by* in situ* perfusion of the liver with collagenase under pentobarbital anesthesia, according to Seglen's method, with a modification by Tanaka et al. [30]. The cells were plated on a 60 mm plastic dish in Williams medium E (WE medium) containing 5% newborn calf serum at 2 × 10^6^ cells/dish and were cultured as monolayers at 37°C in 95% air and 5% CO_2_. After a one-day culture, the cells were incubated for 48 hr in WE medium containing 10^−6 ^M of dexamethasone, 0.1 *μ*g/mL aprotinin, 30 *μ*g/mL kanamycin, and insulin at 10^−10^ to 10^−6 ^M. To examine glutathione concentration- dependent alteration in G6PD activity, buthionine sulfoximine (BSO, a specific inhibitor for glutathione synthesis) was added to the basal incubation medium at a series of concentrations up to 0.5 mM in the presence of 10^−7 ^M insulin. For insulin response to G6PD induction under a restricted glutathione concentration, 0.1 mM BSO was added to the basal medium, and G6PD activity was determined at 10^−10^ to 10^−6 ^M of insulin concentration.

## 3. Results

Liver samples were obtained from rats fed on each of three low protein diets, a 10% SPI, 10% SPI + 3% cystine, and 10% SPI + 3% methionine. Reduced and oxidized glutathione and metallothionein (MT) levels and *γ*-GCS activity in the liver were shown in Table 2. Selective analysis of reduced (GSH) and oxidized (GSSG) forms of glutathione revealed that the SAA-induced increase in the reduced form was prominent and that the GSSG level changed only slightly (Table 2). *γ*-GCS activity, a rate-limiting enzyme of glutathione biosynthesis, was lowered when GSH levels were elevated. Elevation of *γ*-GCS activity in restricted tissue glutathione levels might be a compensative action to raise the levels.

**Figure 1:**
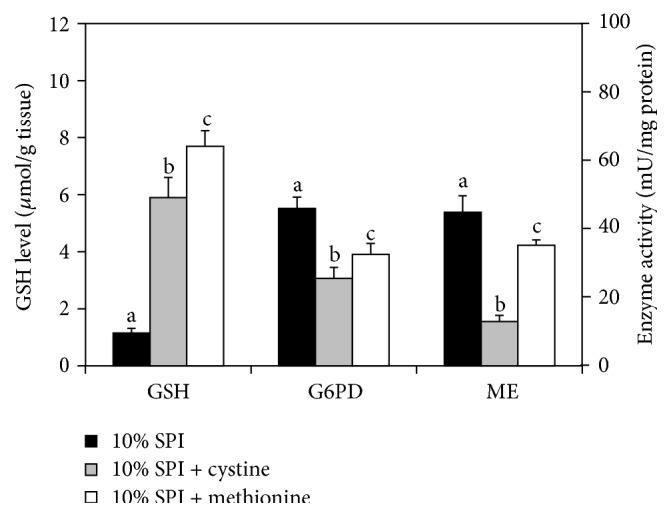
Effects of sulfur-amino acids supplemented to a 10% SPI diet on glutathione concentration and activities of glucose 6-phosphate dehydrogenase (G6PD) and malic enzyme (ME) in rat liver. Values are mean ± SD of 6 rats in each diet group. Different letters show significant difference (*p* < 0.05).

**Figure 2:**
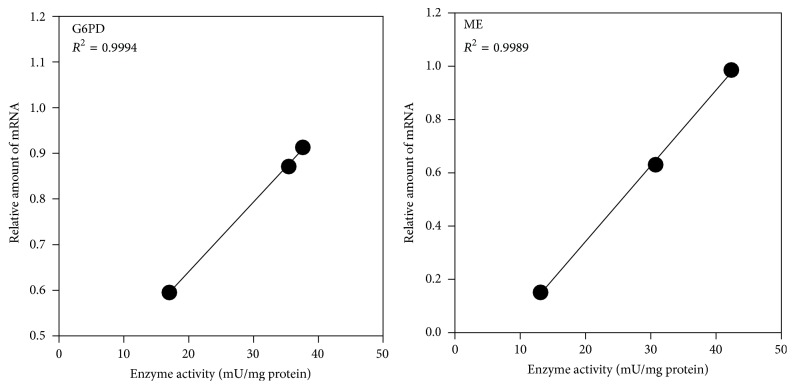
Correlations of liver G6PD and ME activities with their mRNA levels in 10% SPI, 10% SPI + cystine, and 10% SPI + methionine groups. mRNA levels were shown as relative amount to *β*-actin mRNA.

In contrast to the increase in GSH levels, G6PD and ME activities were lowered by supplementing SAA (Figure 1). SAA-induced alterations of G6PD and ME activities were considered to have occurred at the transcriptional levels, since the enzyme activities and their mRNA levels showed reliable correlations (Figure 2).

**Figure 3:**
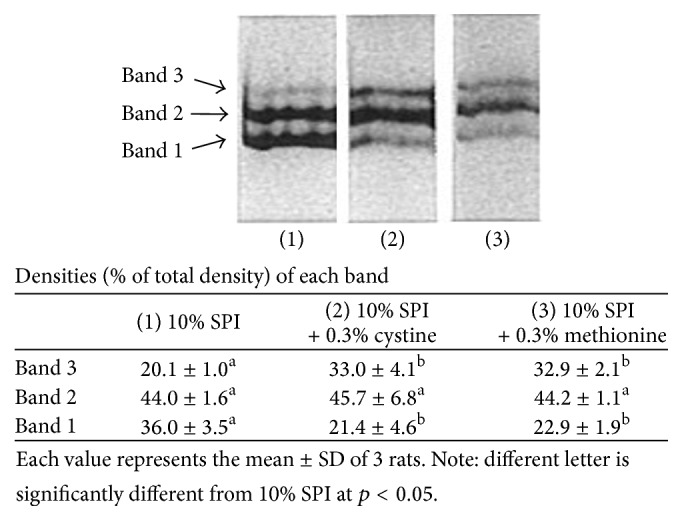
Molecular diversity of liver G6PD separated after electrophoresis on 7.5% polyacrylamide slab gel. Cytosol samples with 5 mU equivalent G6PD activity were applied on the gels and stained for G6PD activity after electrophoresis. Lanes (1), (2), and (3) represent 10% SPI, 10% SPI + cystine, and 10% SPI + methionine, respectively. Bands 1, 2, and 3 represent fully oxidized, partially oxidized, and fully reduced forms. Different letters in bands show significant difference (*p* < 0.05).

G6PD in the liver cytosol is known to exist in 3 dimeric molecular forms: fully reduced, partly oxidized, and fully oxidized, and they can be separated on the polyacrylamide gel electrophoresis as reported by Martins et al. [17]. Liver cytosol samples prepared from rats fed on 10% SPI, 10% SPI + cystine, and 10% SPI + methionine diet were analyzed using a gel electrophoresis, and 3 major bands were obtained as shown in Figure 3. Bands l, 2, and 3 are different molecular forms and are named fully oxidized, partly oxidized, and fully reduced form, respectively [15]. Distribution of the multiple molecular forms of the enzyme was found to be notably different among the diet groups (Figure 3). Although the major components in adult female rats were reported to bands 2 and 3 [17], bands 1 and 2 became the major forms in the 10% SPI group. However, supplementation of SAA to 10% SPI caused significant reduction of band 1, bringing about a distribution similar to the normal pattern reported by Martins et al. [17]. The distribution pattern of the G6PD molecular forms agreed to GSH/GSSG ratio rather than GSSG concentration (Table 2).

**Figure 4:**
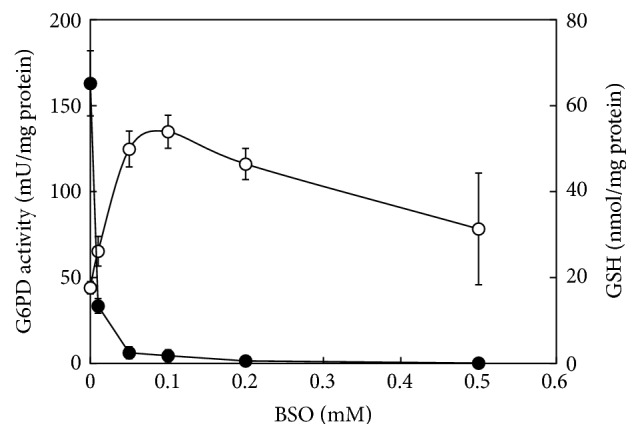
Effect of BSO on glutathione concentration and G6PD activity of isolated cultured hepatocytes. Isolated rat hepatocytes were incubated for 48 h in WE medium containing 10^−6 ^M dexamethasone, 0.1 *μ*g/mL aprotinin, 30 *μ*g/mL kanamycin, and 10^−7^ M insulin in the presence of 0–0.5 mM BSO. Glutathione level (closed circle) and G6PD activity (open circle) were determined at each BSO concentration.

**Figure 5:**
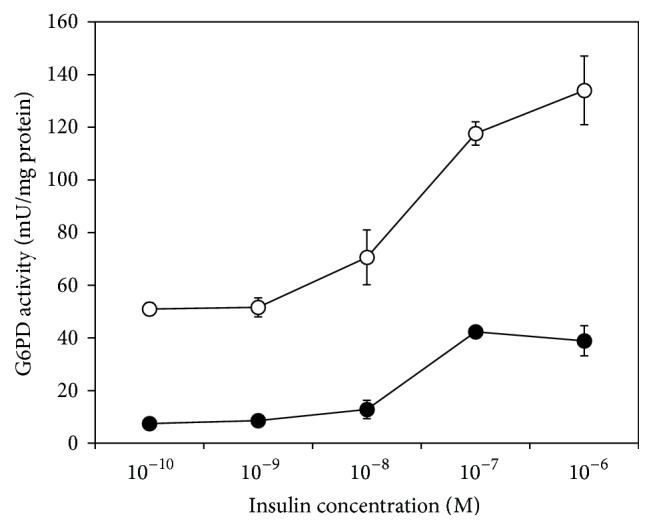
Amplifying insulin response for G6PD activity and effect of BSO. Precultured isolated hepatocytes were incubated for 48 h in the basal WE medium as described in Figure 4. To the basal medium, insulin at a series of concentrations (10^−10^ to 10^−6 ^M) was added, and G6PD activity was determined in the presence (closed circle) and absence (open circle) of 0.1 mM BSO.

Insulin's ability to induce G6PD was examined at different concentrations of GSH using primary culture of the hepatocytes. Cells were incubated with 0.01–0.5 mM BSO, a specific inhibitor of glutathione biosynthesis, in the presence of 10^−7^ M insulin for 48 hr. The GSH concentration-dependent alteration in G6PD activity was depicted in Figure 4. With the increase in the BSO dose, G6PD activity reached its maximum (3-fold of the initial activity) at 0.1 mM BSO with the cellular GSH concentration of less than 5 nmol/mg protein (2.8% of the initial level). It began then to decrease, although the GSH level was kept decreased. Decreased enzyme activity with the higher BSO concentrations would be due to BSO-induced cellular damage, as was made evident from morphological observation using phase contrast microscopy and lactate dehydrogenase activity in the medium (data not shown). Accordingly, a BSO dose level of 0.1 mM was found to be most effective for inducing G6PD activity with minimum cell damage. G6PD activity in the presence of insulin at a series of concentrations (10^−10^ to 10^−6^ M) was determined with and without addition of 0.1 mM BSO. As shown in Figure 5, G6PD activity was elevated with the increased insulin concentration, and the response was remarkably amplified in the presence of BSO. The present results with the isolated hepatocytes came about with the notable increase in G6PD mRNA expression found with Hela cells when an intracellular pool of GSH was reduced [13].

## 4. Discussion 

Laboratory rodents fed on a low protein diet having low SAA, such as 10% SPI, are a useful model for producing low hepatic GSH levels. GSH is an intracellular reducing agent, and low GSH levels eventually caused the greater animal susceptibility to oxidative damage [1, 17]. NADPH, another reducing reagent, is supplied by the action of G6PD and ME. In the present study, we researched the alteration of these enzyme activities in the liver under low GSH levels by feeding rats on a 10% SPI diet. SAA supplementation to 10% SPI caused decreases in G6PD and ME activities concomitantly with increase in GSH level. These results suggested that NADPH production was stimulated under low GSH levels and that NADPH could compensate with GSH as a reducing agent in protecting cells from oxidative stress. A reverse relation between G6PD activity and GSH levels was confirmed also using isolated hepatocytes in the presence of BSO, a specific inhibitor for GSH synthesis.

G6PD was reported to be induced several fold in the presence of 10^−8 ^M insulin in primary cultured hepatocytes [7]. Using cultured hepatocytes in the present study, G6PD activity was amplified through a range of insulin concentrations from 10^−10^ to 10^−6 ^M, and the activity was further augmented by GSH deficiency caused by BSO. mRNA analysis suggested that the induction of G6PD to amplify insulin response by GSH suppression existed prior to and/or at the transcriptional event.

G6PD in rat liver was shown to be separated into 3 dimeric molecular forms on polyacrylamide gel electrophoresis [11]. Bands 1, 2, and 3 shown in Figure 3 represented fully oxidized, partly oxidized, and fully reduced forms, respectively. It was proposed that a shift towards band 1 could be an early event in the degradation of this enzyme, since this form was the most rapidly inactivated by chymotrypsin or microsomes, possibly leading to faster turnover of this enzyme. Although high ratio of band 1 in the 10% SPI group was considered to be due to high oxidative state, GSSG levels were not significantly different among the dietary groups. The GSH concentration and/or GSH/GSSG ratio would have an important role when determining the proportion of the three molecular forms. Under oxidative stress, a considerable portion of an intracellular oxidized form of glutathione was found in protein-bound mixed disulphides, as can be determined after borohydride treatment [31]. In the present experiment, GSH and GSSG were extracted with perchloric acid, but the protein-bound mixed disulfide could possibly have escaped the extraction. Accordingly, the real amount of the oxidized form of glutathione, GSSG plus protein-bound mixed disulfide, would be higher than the level obtained in the present study, especially in 10% SPI-fed rat liver. Faster degradation of G6PD by forming a fully oxidized form would accelerate the turnover rate of the enzyme in rats fed on 10% SPI diet. Despite the accelerated turnover rate, G6PD activity would have been elevated due to increased transcription under restricted glutathione levels in the liver.

A reverse correlation with glutathione levels was also observed in the activity of *γ*-GCS, a rate-limiting enzyme of glutathione synthesis (Table 2). *γ*-GCS activity is well-documented to be induced by various impacts [32–34], but it is feedback inhibited by glutathione itself [35]. Thus, activities of the above two enzymes, G6PD and *γ*-GCS, were regulated by GSH levels but in different manners. G6PD was amplified under low GSH levels via insulin interaction, while *γ*-GCS was controlled by product-binding inhibition. Rise in G6PD activity would allow increase in NADPH to compensate GSH as a reducing agent under increased oxidative status. The inverse correlation between MT, another cytosolic reducing agent, and GSH was found also in this study (Table 2). In GSH depletion, MT levels are elevated to function as a biological defense system against oxidative stress, together with NADPH produced by G6PD.

In conclusion, through animal experiments using rats fed on low protein diet and primary cultured hepatocytes, we found that low GSH levels caused elevation of the hepatic G6PD activity by amplified insulin response for the enzyme expression and accelerated turnover rate of the enzyme molecules by increasing a portion of the fully oxidized form.

## Figures and Tables

**Table 1:** Compositions of the diets.

Component	g/100 g diet
Protein	10.0
Dextrin	15.5
Sucrose	10.0
Soybean oil	5.0
Cellulose	5.0
Mineral mixture (AIN 93)	3.5
Vitamin mixture (AIN 93)	1.0
Choline bitartrate	0.25
^*∗*^Cystine or methionine	0.3
Corn starch	To 100.0

^*∗*^Supplemented to a diet at 0.3%.

**Table 2:** Effect of diet on concentration of glutathione (reduced and oxidized forms), metallothionein (MT), and *γ*-glutamylcysteine synthetase (*γ*-GCS) activity in rat liver.

Diet	GSH (*μ*mol/g liver)	GSSG (nmol/g liver)	GSH/GSSG	MT (nmol Hg/g liver)	*γ*-GCS (mU/mg protein)
10% SPI	1.2 ± 0.2^a^	53 ± 14^a^	22	244 ± 70^a^	9.2 ± 2.4^a^
10% SPI + cystine	5.9 ± 0.4^b^	43 ± 26^a^	137	66 ± 14^b^	5.4 ± 0.9^b^
10% SPI + methionine	7.0 ± 0.6^c^	39 ± 10^a^	179	104 ± 31^b,c^	6.7 ± 1.0^b^

Values are mean ± SD of 6 rats fed on respective diets for 3 weeks.

Activity was expressed as mU/mg protein. Amount of mRNA was expressed as relative intensity. Different letters in the same column denote significant difference (*p* < 0.05).
